# Basal Exposure Therapy: A New Approach for Treatment-Resistant Patients with Severe and Composite Mental Disorders

**DOI:** 10.3389/fpsyt.2016.00198

**Published:** 2016-12-19

**Authors:** Didrik Heggdal, Roar Fosse, Jan Hammer

**Affiliations:** ^1^Division of Mental Health and Addiction, Vestre Viken Hospital Trust, Lier, Norway

**Keywords:** treatment resistance, severe mental illness, basal exposure therapy, self-regulation, evaluation study

## Abstract

New treatment approaches are needed for patients with severe and composite mental disorders who are resistant to conventional treatments. Such treatment-resistant patients often have diagnoses of psychotic or bipolar disorders or severe personality disorders and comorbid conditions. In this study, we evaluate basal exposure therapy (BET), a novel ward-integrated psychotherapeutic approach for these patients. Central to BET is the conceptualization of undifferentiated existential fear as basic to the patients’ problem, exposure to this fear, and the therapeutic platform complementary external regulation, which integrates and governs the totality of interventions throughout the treatment process. BET is administered at a locked-door ward with 6 patient beds and 13.5 full-time employees, including a psychiatrist and 2 psychologists. Thirty-eight patients who had completed BET were included, all but two being female, mean age 29.9 years. Fourteen patients had a diagnosis of schizophrenia or schizoaffective disorder (F20/25), eight had bipolar disorder or recurrent depressive disorder (F31/33), eight had diagnoses in the F40–48 domain (neurotic, stress-related, and somatoform disorders), five were diagnosed with emotionally unstable personality disorder (F60.3), and three patients had other diagnoses. Twenty of the patients (53%) had more than one ICD-10 diagnosis. Average treatment time in BET was 13 months, ranging from 2 to 72 months. Time-series data show significant improvements in symptoms and functioning from enrollment to discharge, with effect sizes at 0.76 for the Dissociation Experience Scale, 0.93 for the Brief Symptom Inventory, 1.47 for the Avoidance and Action Questionnaire, and 1.42 and 1.56, respectively, for the functioning and symptom subscales of the Global Assessment of Functioning Scale. In addition, the patients used significantly less antiepileptic, antipsychotic, anxiolytic, and antidepressant medications at discharge than at treatment enrollment. Patient improvement across treatment was associated with the following duration of time in BET, the successful completions of the exposure component of BET, positive changes in experiential avoidance as measured with the Acceptance and Action Questionnaire, and high symptom levels and low levels of functioning at treatment start. The findings indicate that BET may be a promising inpatient psychotherapeutic approach for previously treatment-resistant patients with severe and comorbid conditions.

## Introduction

Patients who do not respond to repeated treatments represent a particularly demanding challenge for specialized mental health-care services. These patients have a perpetual need for continued care and protection, and the toll on patients and their families in terms of suffering and distress is staggering (1–3). For society, the cost related to loss of life from suicide, increased drug and alcohol abuse, loss of productivity, and increase in overall health costs is almost unfathomable (4–6). This article focuses on a diagnostically many-faceted subgroup of treatment-resistant patients, presenting with schizophrenia spectrum disorders, dissociative disorder, bipolar disorder, or complex post-traumatic stress disorder (PTSD), often with a diagnosis of one or more comorbid personality disorders. The typical clinical picture comprises delusions, hallucinations, dissociation, and mood fluctuations, more often than not in combination with deliberate self-harm and suicide attempts.

Causes for treatment resistance are still unclear, although poor insight into the need of treatment may play a role (7, 8). Treatment-resistant patients in all the noted diagnostic domains often have particularly high symptom loads, comorbid conditions, more impaired psychosocial functioning including unemployment and dependent living conditions, more severe negative symptoms and cognitive deficits, more self-injurious behavior, lower quality of life, and more often substance abuse and aggressive behavior than other patients (2, 9–12).

The patients’ continued suffering and the extensive cost following functional impairment make it imperative to develop novel and effective treatment approaches. One such possible approach is basal exposure therapy (BET), which during the last decade has been developed and implemented within the framework of ordinary clinical care at a closed inpatient ward at Vestre Viken Hospital Trust in Norway (13). In contrast to symptom-oriented treatments, the BET modality has a main focus on mobilizing the patient’s resources and promotion of autonomy. Throughout its developmental phase, BET has been continually evaluated with a naturalistic observational study design. This article presents the first quantitative study on the effects of BET. Before we describe the BET modality, we summarize existing evidence from controlled studies, primarily randomized controlled trials, on treatment options for this treatment-resistant patient group.

### Controlled Evidence of Effective Approaches

Patients with schizophrenia who do not respond to recommended doses of antipsychotic medication have received considerable interest in the scientific literature. Only sporadic attention has been paid to treatment-resistant patients with other diagnosis, including bipolar disorder, severe and composite personality disorders, and dissociative disorders.

In general, the majority of treatment attempts for the patient group have been psychopharmacological, often poly-pharmacy that has not been covered by pharmacological guidelines (14) or other biological interventions, e.g., electroconvulsive therapy, transcranial magnetic stimulation, transcranial direct current stimulation, and deep brain stimulation (11). A review of 280 available RCT studies for treatment-resistant schizophrenia concluded that there is a paucity of trials for most types of therapeutic approaches. The exception is clozapine, but with only modest effects indicated (15). A small set of studies evaluated cognitive behavioral therapy (CBT) for non-responding schizophrenic patients, but without finding positive effect (16). We identified only one controlled study that reported positive effects for a psychotherapeutic approach. In this study, patients with “chronic schizophrenia” who were on maintenance medication exhibited a small improvement in community functioning and symptoms of auditory hallucinations after receiving cognitive adaptation training compared to CBT and treatment as usual (17).

A review of the best-quality RCT studies for treatment-resistant bipolar disorder/bipolar depression identified only seven trials, five medical treatments and two electroconvulsive therapy treatments (18). In concluding the review, Sienaert et al. (18) characterized the current status of treatments for these patients as “experimental only.” A small set of other approaches have been described, including behavioral therapy, sleep deprivation, light therapy, and CBT but with effects that appear to be limited (19). We found no RCT studies for treatment-resistant dissociative disorders or (composite) personality disorders.

Suggestive evidence has been presented in a small set of studies based on non-randomized trials and case studies. Chakhssi et al. (20) reported small-to-moderate decreases in general psychopathology and pathological personality traits by both acceptance and commitment therapy and CBT for patients with personality disorders who had not responded to outpatient treatment. In a case-control study, Bales et al. (21, 22) found improvement after mentalization-based therapy on several measures in a cohort study of treatment-resistant patients with borderline personality disorder and comorbid conditions, the latter including other personality disorders, anxiety disorders, mood disorders, eating disorders, and substance abuse disorder. Chlebowski and Gregory (23), using dynamic deconstructive psychotherapy, reported nominal reductions in dissociative scores in three patients with combined dissociative identity disorder and borderline personality disorder, a comorbid condition where no evidence-based treatment exists.

### Basal Exposure Therapy

Basal exposure therapy was developed out of a need for novel treatments for low-functioning patients with severe and comorbid conditions who had not benefited from multiple prior treatment attempts. The BET approach originated with hypothesizing two main pathological mechanisms that cross diagnostic categories and may generate symptoms and undermine patients’ capability to regulate emotion and behavior. First, at a phenomenological level, recurrent observations seem to indicate that these patients suffer an impending existential anxiety. This anxiety appears to be associated with and trigged by affective arousal, which instigates experiential avoidance (EA) (24, 25). The habit to avoid may maintain and reinforce the patient’s problems (26, 27). Over time, the fear of affective arousal may evolve into a persistent phobic condition, which in BET is labeled *basal phobia* or *existential catastrophe anxiety*. Basal phobia contrasts qualitatively with distinct affect phobias, e.g., fear of guilt, anger, and sadness (28). Explorations indicate that this basal phobia typically is experienced as fear of disintegrating or falling apart, being engulfed by total emptiness or stuck in eternal pain (29). The desperation to use whatever means available to terminate affective arousal and avoid the impending catastrophe often seems to leave these patients with suicide as the ultimate choice. Second, at an interpersonal level, the patients’ treatment histories are typically characterized by the health-care system’s repetitive efforts to regulate symptoms and behavior by imposing high levels of restriction, excessive use of psychopharmacological interventions and coercive measures such as forced medication, and physical and mechanical restraints. The individual response to excessive external regulation may depend on the patient’s typical attachment strategies (30, 31). Some patients in the BET program report that health-care workers’ efforts to impose control increased opposition and hostility, while others reacted with despair and helplessness (32).

With reference to these pathological mechanisms and with an ambition to reinstate the patients’ own capability to regulate emotion and behavior, two main components of the BET model were developed. These are, first, BET as a psychotherapeutic approach and distinct exposure procedure and, second, the autonomy-promoting strategy complementary external regulation (CER) (32). CER is a contextual calibration tool with the intended function to prevent and reverse pathology-maintaining interactions between the patient and health professionals. This strategy represents the coordinating platform from which the total body of psychotherapeutic and milieu therapeutic interventions are organized and administrated 24/7 throughout the treatment process.

Basal exposure therapy can be seen as a second-order change intervention aiming at helping the patients to relate to pain and fearful inner experiences instead of using problem-maintaining avoidance strategies (33). In BET, learning to relate to pain as a part of life is more or less caricatured and taken to extremes. However, radical acceptance of the most aversive and frightening inner experiences, i.e., the existential catastrophe anxiety, is hypothesized within the BET approach to augment these patients’ treatment response considerably. Within this ambition, BET aims at a process-oriented use of medication, where medication is reduced concomitant with patient improvement. Hence, CER-supported psychotherapy is the primary intervention, while medication is secondary.

### Aims

We used a naturalistic within-subject time-series design to evaluate changes in symptoms and functioning for the first 38 patients who have completed BET and provided evaluation data. We also aimed at testing the involvement of central-specific therapeutic features in BET and the role of psychopharmacological treatment. Our research hypotheses were as follows: (1) at discharge compared to enrollment to BET, patients exhibited lower levels of symptoms and higher levels of functioning; (2) the patients’ use of psychopharmaca is reduced across treatment, with no “compensatory” increase in the use of alcohol or drugs; (3) reduced EA across the treatment course is associated with improvements in symptoms and functioning; (4) improvements in symptoms and functioning are associated with the degree to which the exposure component of BET was successfully completed; and (5) those patients with the lowest level of symptoms and the highest level of functioning at discharge tend to be those who exhibit the lowest levels of dissociation and EA. In addition, we examined (6) the influence of time spent in BET on improvement in symptoms and functioning.

## Materials and Methods

Basal exposure therapy is implemented at an inpatient ward for psychotic and complex disorders at Vestre Viken Hospital Trust in Eastern Norway. The hospital trust provides specialized mental health services for a community population of approximately 470,000. Patients are admitted to the ward from other clinical units covered by the hospital trust as well as from other hospital trusts. Since BET represents a contrasting alternative to inpatient approaches that to a larger extent focus on symptom reduction, a priority at the outset has been to establish a naturalistic and systematic evaluation frame. This framework allows for within-subject time-series analysis of changes in patient symptoms and functioning across the treatment course.

### Participants

The inclusion criteria to BET are (1) persistently low, falling or dramatically fluctuating levels of psychosocial functioning characterized by Global Assessment of Functioning (GAF) Scale scores below 35; (2) diagnosis of schizophrenia spectrum disorders, bipolar disorder, PTSD, dissociative disorder, or composite personality disorder; and (3) prior outpatient and inpatient treatment histories, with adequate treatments but no lasting improvement. The treatment histories often have been characterized by excessive symptoms that were either manifest or fluctuating and combined with high levels of turmoil and/or deep resignation. Patients are excluded when the personality is dominated by persistent hostility, when they are developmentally challenged (IQ below 70), and when they have a history of extensive substance abuse combined with violent behavior and pronounced cognitive impairment. Patients with a brain organic disorder are excluded, with the exception being such patients who for years are not handled by other parts of the treatment system and remain extensive users of inpatient services. Moreover, patients are excluded when they present exclusively with an unequivocal personality disorder diagnosis, typically the emotionally unstable type, following guidelines that these patients should not receive long-term treatment at inpatient wards (34). The exception is when such patients have particularly long and turbulent treatment histories with repetitive and dramatic suicide attempts, resulting in prolonged hospital stays and the excessive use of force.

By using these criteria, to date, 49 patients have been enrolled to BET. Six of these patients are currently in treatment. Two former patients decided to end BET before treatment completion, one died by overdose of illegal drugs, and two were transferred to other types of hospital treatments when it became clear that the exclusion criterion of persistent hostility was encountered. The participant group available for this publication consists of 38 patients, mean age 29.9 years (SD = 8.1). All but two patients were female. This gender bias may reflect the organization of the services in the hospital trust. While low-functioning female patients who present with chronic suicidality typically are referred to BET, their male counterparts with higher levels of hostility and antisocial characteristics usually are transferred to high-level security wards or other psychosis wards.

The patients were diagnosed by use of the ICD-10 diagnostic system (35). For the study, we used the diagnoses set by the referring institutions, which are presented in Table 1.

**Table 1:** **Diagnoses at admission to basal exposure therapy**.

ICD-code	Main diagnosis	*n*	*n* with comorbid disorder	Comorbid disorders in subgroup
F20/25	Schizophrenia	8	5	Alcohol dependence, other psychoactive substance dependence, post-traumatic stress disorder (PTSD), emotionally unstable personality disorder
	Schizoaffective disorder	6		
F31/33	Bipolar affective disorder	4	7	Alcohol dependence, poly-substance drug use, social phobias, obsessive-compulsive disorder, PTSD, anorexia nervosa, bulimia nervosa, paranoid personality disorder, emotionally unstable personality disorder, personality disorder unspecified, dependent personality disorder, enduring personality change after catastrophic experience, disturbance of activity and attention
	Recurrent depressive disorder	4		
F40–49	Obsessive–compulsive disorder	4	6	Major depressive episode, recurrent depressive disorder, phobic anxiety disorders, PTSD, anorexia nervosa, emotionally unstable personality disorder, avoidant personality disorder, personality disorder unspecified, mixed and other personality disorders
	PTSD or dissociative disorder	4		
F60.3	Emotionally unstable personality disorder	5	2	Alcohol abuse, major depressive episode, persistent mood (affective) disorders, phobic anxiety disorders, anorexia nervosa, bulimia nervosa
Other		3^a^		

*^a^One of these patients was diagnosed with psychotic disorder*.

Average treatment time in the BET program for the 38 patients was 13 months, ranging from 2 to 72 months. All patients previously had both outpatient and inpatient treatments, with a mean of 8.0 years (SD = 5.9 years) since the first admittance to inpatient treatment in specialized mental health care. All patients had multiple (at least two) previous treatment attempts with psychopharmaca adequate to main diagnosis.

### The BET Program

Basal exposure therapy is administered at a locked-door ward with 6 patient beds and 13.5 full-time employees, including a psychiatrist and two psychologists. The program is treatment intensive and strictly organized. In the study period, duration of treatment time in BET was individually customized to each patient’s needs. Each patient has a treatment team with a team coordinator, a psychologist or psychiatrist, and two or three trained co-therapists (nurses or other milieu therapists). There is one daily individual therapy session with one of the therapists, who take turns to vary the contextual components and prevent the process from becoming static. In addition, there are 5-min focus sessions every morning and evening. These sessions are used, respectively, to identify and formulate a specific challenge for the day and to identify and reflect on what the patient has learned. The purpose of framing the therapeutic workday with focus sessions is to increase the learning effect and promote commitment to treatment. The patients participate in their own treatment and team meetings and in a weekly psycho-educative group (not group therapy), which once a month is led by a former BET patient. In most cases, there are two to three sessions with close relatives in the treatment process to eliminate or modify the effects of pathology maintaining factors in the patient’s social relationships. Likewise, routine meetings with the general practitioner, outpatient therapist, and community health services ensure that follow-up treatment supports patient autonomy.

When a patient is admitted to the BET program, the first task of the treatment team is to facilitate cooperation and compliance with the program’s treatment goals. Initially, the main strategy is systematic psychoeducation combined with the therapeutic stance inherent in CER. By putting into practice the existential postulate that all human beings are free and create their own lives by deliberate choices and actions (36), CER seeks to reallocate responsibility and locus of control. CER alternates between two sets of opposite contextual approaches. The primary CER regime is under-regulation, which is characterized by a general normalization of all interaction. Examples here are that the patients on the one hand are free to do what they want, e.g., go for a walk whenever they feel like going for a walk, and on the other hand, that they are expected to be accountable when it comes to keeping appointments they make and to inform the staff if they change their plans. In the under-regulation regime, validation and solution-focused interventions are used to optimize and consolidate autonomous functioning (37, 38). The back-up regime, over-regulation, is a motivating standstill with strict focus on safety. Over-regulation is used only if the patients repeatedly fail to obtain self-regulation and life and health are at risk. However, this regime is not characterized by the use of force but by validating care and a focus on the patient’s situational options and actions. The treatment team is awaiting the patient’s initiative to a “coping-dialog.” When he or she have described coping strategies that may be functional alternatives to acting out or self-destructive behavior and have expressed motivation and willingness to try out these strategies, the regime is switched back to under-regulation. CER may metaphorically be denoted as a “secure base,” borrowing Bowlby’s (39) term for the relational platform that children need in order to follow the normal path of development. To enable therapists and staff to adjust the administration of CER to the individual patient’s attachment strategies, BET has included basic elements from the dynamic-maturational model (DMM) (30, 40). In DMM-informed CER, potential pitfalls associated with regression are predicted and detected and then averted by various strict forms of validating interventions. The treatment team continuously monitors patients’ regressive and progressive responses. If indicated, the two regimes are switched back and forth to facilitate progressive responses.

When a sufficiently stable working alliance is established, the next objective, and the core intervention in BET, is to replace excessive avoidance of existential catastrophe anxiety with exposure and acceptance. First, patient and therapist explore and identify the patients’ typical avoidance behaviors, including physical behaviors, body posture, restricted respiration, the way he or she talks, and mental maneuvers like intellectualization and externalization. Then the patient is invited to expose himself or herself by choosing not to avoid, i.e., refrain from doing what he or she usually does to keep affective arousal at a bearable level. By this therapeutic procedure, the patient realizes step by step, and also by flooding experiences (41), that what used to be experienced as existential threats may be unpleasant and painful, but no longer dangerous. When the patient begins to choose exposure as opposed to avoidance, solution-focused interventions (38) are used to reinforce and consolidate self-exposure and self-regulating skills and consequently self-efficacy and empowerment.

### Measures and Procedures

We used the split version of the GAF scale to assess general functioning and symptoms. GAF constitutes Axis V in DSM-IV-TR (42) and aims at global psychological, social, and occupational characteristics. The reliability of GAF is considered improved when several raters are used (43). We determined GAF as consensus scores between two or three trained clinicians based on clinical observations on the ward from the last week. All raters were trained according to national procedures (44).

Patients scored their own symptoms with the Brief Symptom Inventory (BSI), which consists of 53 items scored on 5-point Likert scales. The BSI includes nine subscales: somatization, compulsiveness, interpersonal sensitivity, depression, anxiety, hostility, paranoid thoughts, and psychoticism. In the analysis, we used the Global Severity Index (GSI), defined as the mean score across the 53 items. BSI (and GSI) has good psychometric properties, with Cronbach’s alpha between 0.71 and 0.85 (45).

Dissociation was measured with the Dissociation Experiences Scale (DES) (46). DES is a self-rating form that measures the degree of experienced dissociation and consists of 28 items. Each item is scored with respect to the proportion of time that the dissociative symptom is experienced, ranging from 0% (never) to 100% (always). DES has test-retest reliability at 0.84–0.96 and Cronbach’s alpha at 0.95 (46, 47).

Experiential avoidance was assessed using the Acceptance and Action Questionnaire (AAQ). AAQ is a self-rating form originally designed to measure EA in terms of psychological flexibility vs. inflexibility (24). Low EA is associated with the use of second-order coping strategies, which is a central concept and the primary treatment goal in BET (33). We used the original nine-item version of the AAQ, which has adequate criterion-related, predictive, and convergent validities^1^. We had the AAQ translated to Norwegian following standard procedures.

Patients rated their own use of alcohol and drugs with the Alcohol Use Disorder Identification Test (AUDIT) (48) and Drug Use Disorders Identification Test (DUDIT) (49). The AUDIT includes 10 questions about hazardous and harmful patterns of alcohol use and dependence. The DUDIT includes 11 questions about harmful use or abuse of a list of drugs. Both instruments have satisfactory psychometric properties in clinical and non-clinical samples, with overall reliability above 0.80 and convergent validity, sensitivity, and specificity above 85% (50–53).

Information on the use of medications was extracted from the patients’ medical charts in the electronic patient journals. We focused on regular medications (and doses) at enrollment to BET and at discharge for the following five WHO-defined categories: N03A antiepileptics, N05A antipsychotics, N05B anxiolytics, N05C hypnotics, and N06A antidepressants. We transformed the dose of each medication that the patients had been administered into defined daily doses (DDD), according to WHO guidelines (http://www.whocc.no/atc_ddd_index/), and summed together DDDs for different drugs that belonged to the same category.

Exposure to distressing and aversive thoughts and feelings is a core therapeutic component of BET. However, the degree to which patients have completed exposure has varied, reflecting the degree to which a productive working alliance has been established, i.e., whether the patient hesitates or actively commits herself/himself and engages in exposure therapy. We quantified the exposure component of BET on a 0 to 3 scale, with 0 representing no exposure, 1 some degree of/intermittent exposure, 2 systematic work with graded exposure, and 3 flooding (trials of full exposure). Two clinicians, who knew all the patients and their therapeutic processes well, independently used the exposure scale to score each patient. For 29 of the 38 patients (76%), the 2 raters gave the same score. For the nine patients with deviant scores, the raters subsequently reached a consensus decision. For the statistical analysis, we dichotomized the variable and sorted patients scored at 0 or 1 into a “low-exposure” group and those scored at 2 or 3 into a “high-exposure” group. On this dichotomized variable, the two raters initially agreed on the exposure score for 37 of the 38 patients (97%).

### Statistical Analyses

The scores on GAF, GSI, DES, and AAQ were normally distributed, and changes from enrollment to discharge from BET were analyzed with paired sample *t*-tests. Effect sizes (Cohen’s *d*) were calculated for each measure, using test statistics from the dependent *t*-test (*t, r, n*), see http://www.psychometrica.de/effect_size.html. The use of medications was positively skewed for all medication categories. The same was the case for scores on AUDIT and DUDIT. Changes in these variables from enrollment to discharge were analyzed with Wilcoxon signed-rank test for related samples.

To analyze the possible contribution of changes in EA, measured with AAQ, on changes in GAF, GSI, and DES from enrollment to discharge, we first calculated changes in AAQ scores for each patient. The resulting variable was dichotomized into a “low-change” group who changed at or below the median (less than or equal to nine points on the AAQ) and a “high-change” group who changed above the median. We then used independent sample *t*-tests to analyze if AAQ group (low, high) had an effect on changes in the outcome measures. Likewise, the contribution of the exposure component of BET on changes in the outcome measures was analyzed with independent sample *t*-tests, using exposure level (low vs. high) as dichotomized independent variable.

Pearson correlation analysis was used to investigate the association between time spent in BET and changes in the outcome measures from enrollment to discharge. For outcome measures that were significantly associated with time in BET, we continued with regression analysis, using time in BET and degree of exposure (low, high) as predictors, and, as dependent variable, change in the outcome measure.

Next, we used Pearson correlation tests to analyze whether, at discharge, lower scores on DES and higher scores on AAQ were associated with low symptom and high functioning scores (GAF and GSI). In a final series of analysis, we tested whether patient characteristics at enrollment could predict scores on the outcome measures at discharge. Independent sample *t*-tests were used to test whether two diagnostic categories, schizophrenia and other psychosis with or without comorbid conditions (“psychosis”) (*n* = 15) vs. non-psychotic, comorbid conditions (“non-psychotic”) (*n* = 23) (see Table 1) were differentially associated with changes in the outcome measures. To analyze whether scores on the outcome measures at enrollment were associated with changes in the same measures from enrollment to discharge, we used Pearson *r*.

Outcome data were available for all patients only for GAF. Patients with missing data on a given variable were excluded from all analyses where this variable was used. The analyses were performed in SPSS version 23.

### Ethics Statement

The Regional Committees for medical and health research ethics has considered this evaluation study as a systematic self-evaluation of clinical practice outside their mandate. The Data Protection Office for Research in Vestre Viken has approved publishing the data. The Data Protection Office stated that the topic is of great public interest, and informed consent was not deemed necessary because it would be impossible to identify the individual patients from the aggregated data analyzed in the study.

## Results

The patients’ ICD-10 diagnoses at enrollment to BET are summarized in Table 1. The 38 patients spent an average of 13.1 months (SD = 14.4) in BET.

### Changes from Enrollment to Discharge

**Figure 1:**
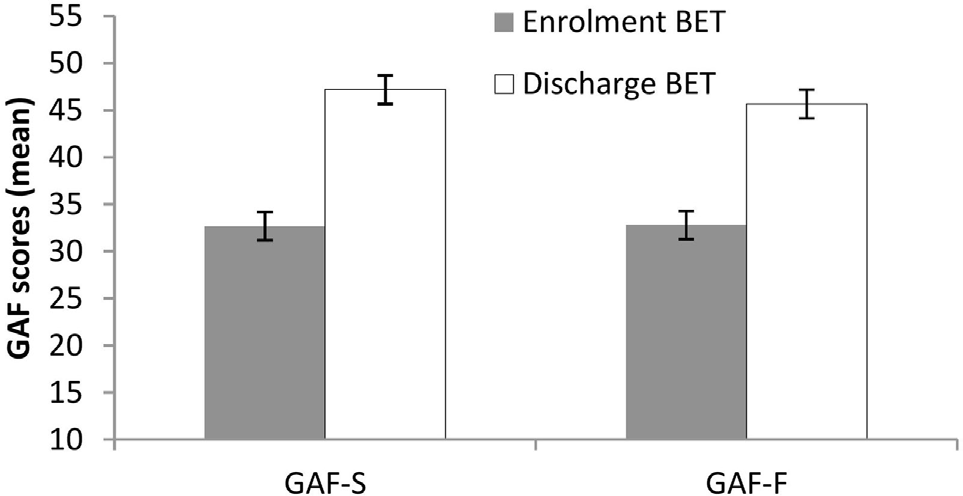
**Changes in Global Assessment of Functioning from enrollment to discharge**. Lines are SEs.

Significant changes (improvements) from enrollment to discharge were seen in both GAF-S, mean 32.7 vs. 47.2, *t*(37) = 6.5, *p* < 0.001, and GAF-F, mean 32.8 vs. 45.7, *t*(37) = 6.2, *p* < 0.001 (Figure 1). Effect sizes (Cohen’s *d*) were large: 1.56 for GAF-S and 1.42 for GAF-F.

**Figure 2:**
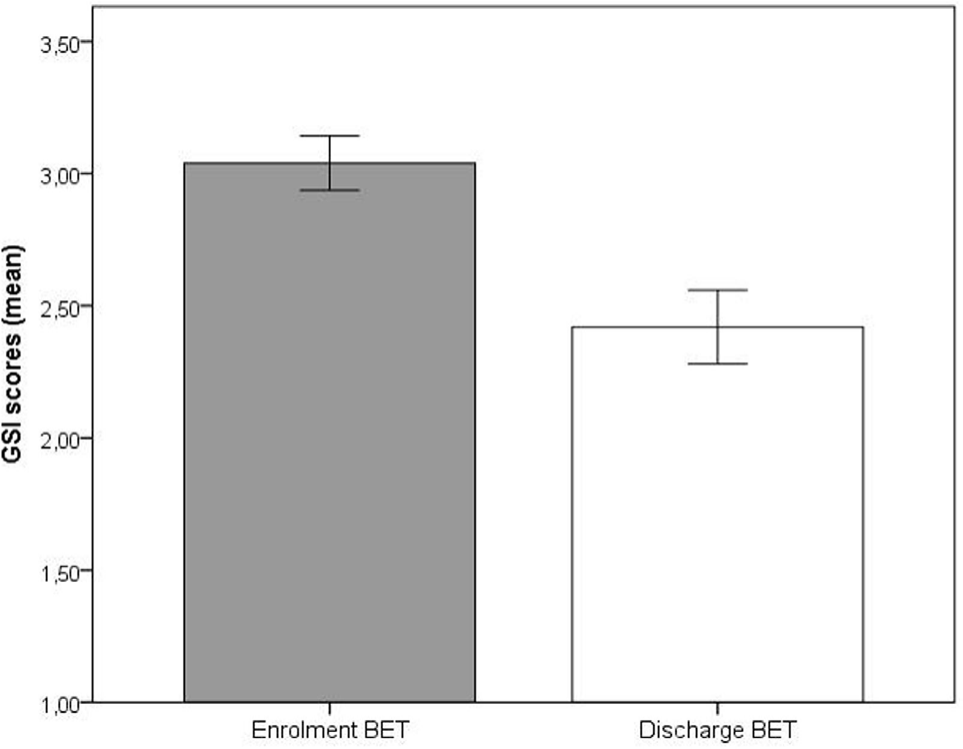
**Changes in Global Severity Index from enrollment to discharge**. Lines are SEs.

**Figure 3:**
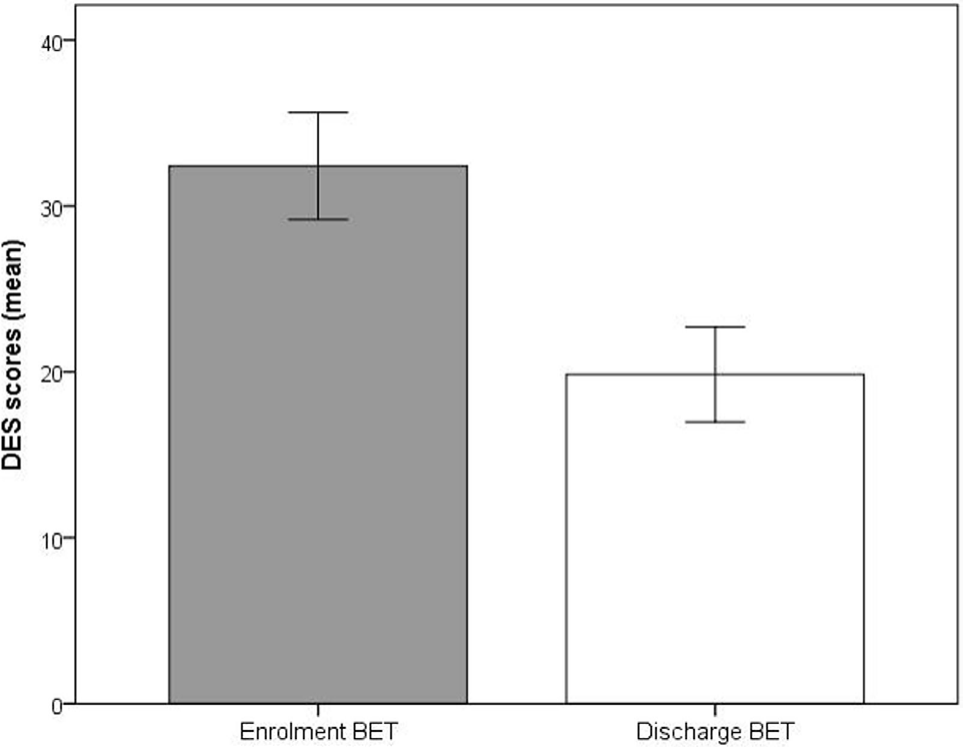
**Changes in Dissociation Experience Scale from enrollment to discharge**. Lines are SEs.

**Figure 4:**
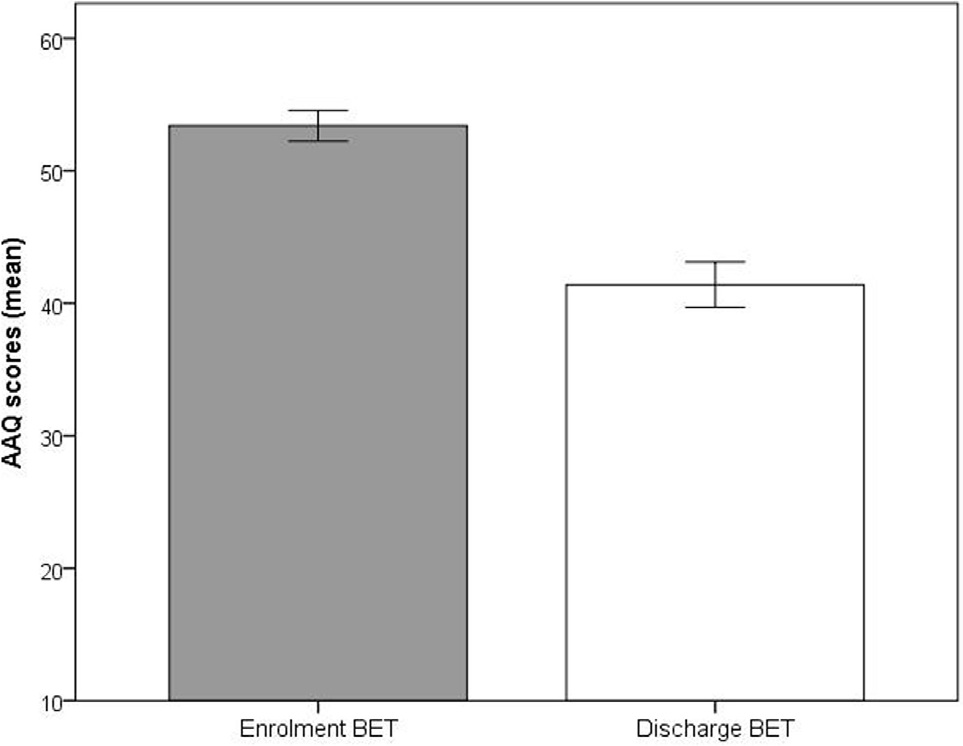
**Changes in experiential avoidance (Acceptance and Action Questionnaire) from enrollment to discharge**. Lines are SEs.

Significant improvements (*p* < 0.001) at discharge compared to enrollment to BET were also seen for GSI, mean 3.0 vs. 2.4, *t*(28) = 4.5, *p* < 0.001 (Figure 2), DES, mean 32.7 vs. 19.8, *t*(28) = 4.9, *p* < 0.001 (Figure 3), and AAQ, mean 52.5 vs. 41.4, *t*(29) = 7.3, *p* < 0.001 (Figure 4). Effect sizes were *d* = 0.93 for GSI, *d* = 0.76 for DES, and *d* = 1.47 for AAQ.

**Figure 5:**
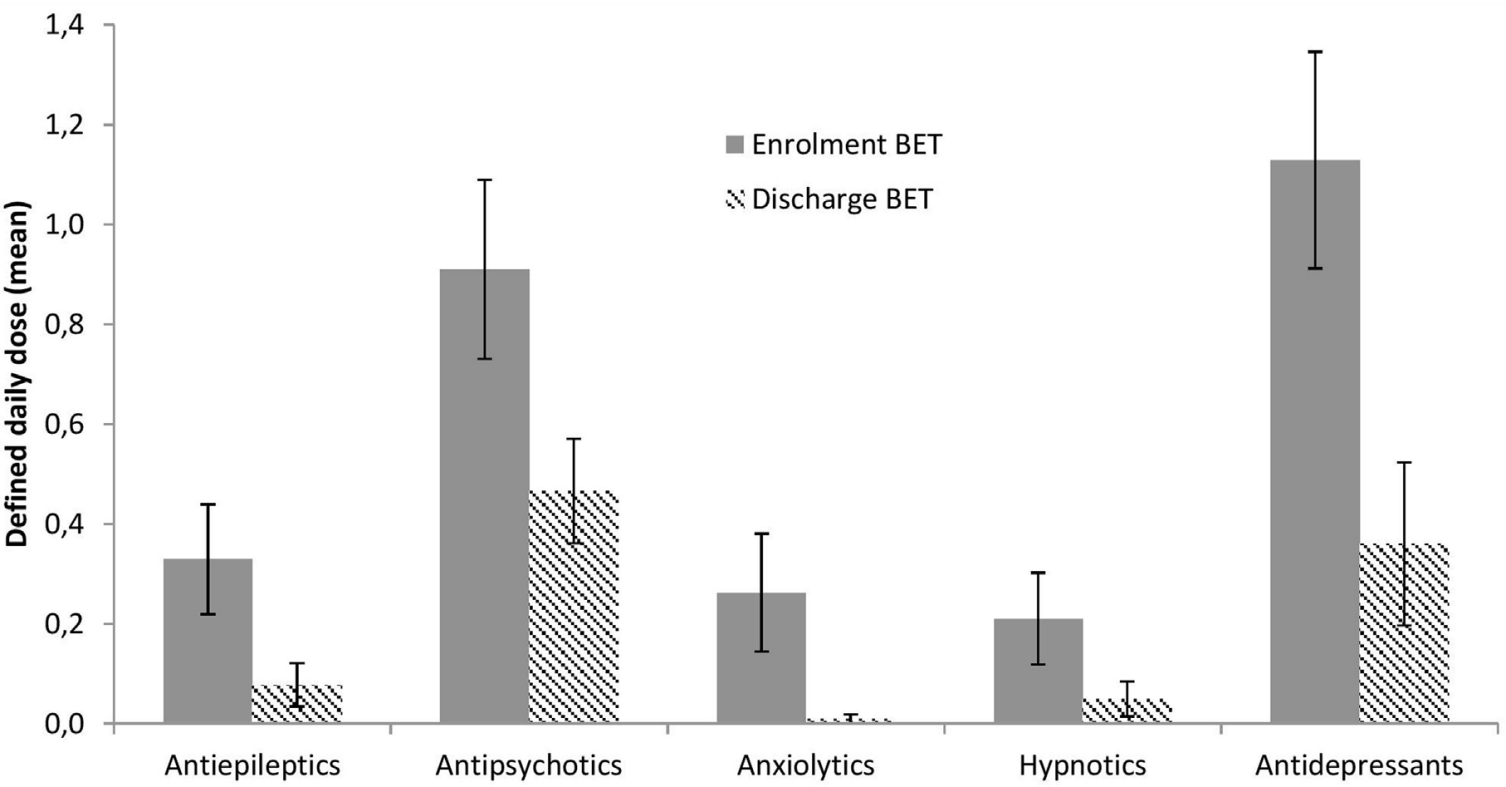
**Changes in psychopharmacological treatment from enrollment to discharge**. Lines are SEs.

The use of medications at enrollment and discharge from BET is illustrated in Figure 5 and depicted in more detail in Table 2. Significant reductions across treatment were seen for antiepileptics, *p* = 0.033, antipsychotics, *p* = 0.002, anxiolytics, *p* = 0.028, and antidepressants, *p* < 0.001, with a similar trend for hypnotics, *p* = 0.066.

**Table 2:** **Use of regular medications at admission and discharge**.

ICD-code	Medication type	Admission		Discharge	
		*n*	*M* DDD^a^ (SD)	*n*	*M* DDD^a^ (SD)
F20/25 (*n* = 14)	Antiepileptics	3	0.95 (0.62)	2	0.9 (0.14)
	Antipsychotics	12	1.62 (1.34)	9	1.0 (0.38)
	Anxiolytics	3	2.17 (1.04)	0	–
	Hypnotics	3	1.67 (0.58)	0	–
	Antidepressants	6	2.04 (1.25)	2	1 (0)
F31/33 (*n* = 8)	Antiepileptics	4	0.83 (0.45)	0	–
	Antipsychotics	6	0.55 (0.43)	3	0.44 (0.35)
	Anxiolytics	1	0.9 (0)	0	–
	Hypnotics	0	–	0	–
	Antidepressants	4	1.1 (0.57)	0	–
F42–44 (*n* = 8)	Antiepileptics	1	2.67 (0)	0	–
	Antipsychotics	4	1.58 (0.68)	2	1.54 (1.0)
	Anxiolytics	1	0.3 (0)	0	–
	Hypnotics	1	1 (0)	1	0.67 (0)
	Antidepressants	6	2.21 (1.42)	3	2.93 (1.68)
F60.3 (*n* = 5)	Antiepileptics	2	1.17 (0.71)	1	0.83 (0)
	Antipsychotics	4	0.85 (0.43)	3	0.9 (0.74)
	Anxiolytics	1	0.92 (0)	0	–
	Hypnotics	1	1 (0)	1	1
	Antidepressants	3	2.25 (0.9)	0	–
Other (*n* = 3)	Antiepileptics	0	–	0	–
	Antipsychotics	0	–	0	–
	Anxiolytics	1	0.3	1	0.3
	Hypnotics	0	–	0	–
	Antidepressants	2	0.88 (0.18)	2	0.75 (0.35)

*^a^Mean defined daily doses (DDD) for those who used the medication. Four participants had missing data*.

No significant changes from enrollment to discharge were seen for AUDIT, mean 5.2 vs. 4.5, *p* = 0.43, or DUDIT, mean 3.0 and 3.4, *p* = 0.73.

### Analysis of Therapeutic Mechanisms

As shown in Table 3, participants with high improvements compared to those with low improvements in EA (AAQ) had significantly higher improvements on GAF-S, *p* = 0.002 and GAF-F, *p* = 0.005, but not on GSI or DES.

**Table 3:** **Changes in outcome measures as a function of low and high changes on the Acceptance and Action Questionnaire (AAQ)**.

Outcome measure	Mean changes		*t* Value (df)	*P* value
	Improvement on AAQ: low	Improvement on AAQ: high		
GAF-S	+6.5	+21.5	3.6 (28)	0.002
GAF-F	+5.7	+16.3	3.1 (28)	0.005
BSI: GSI-score	−0.46	−0.79	1.21 (27)	0.24, ns
DES	−11.5	−13.5	0.37 (27)	0.72, ns

*GAF, Global Assessment of Functioning, symptom (S) and functioning (F) subscales; GSI/BSI, Global Severity Index from the Brief Symptom Inventory; DES, Dissociation Experience Scale*.

As seen in Table 4, patients with a high degree of completion compared to those with a low degree of completion of the exposure component of BET had significantly larger improvements on GAF-S, *p* = 0.025, GAF-F, *p* = 0.008, and GSI, *p* = 0.030, with a similar trend for DES, *p* = 0.055.

**Table 4:** **Changes in outcome measures as a function of low and high completion of the exposure component of basal exposure therapy**.

Outcome measure	Mean changes		*t* Value (df)	*P* value
	Low exposure	High exposure		
GAF-S	+9.9	+19.7	2.3 (36)	0.025
GAF-F	+7.8	+18.5	2.8 (36)	0.008
BSI: GSI-score	−0.32	−0.90	2.3 (27)	0.030
DES	−7.5	−17.3	2.0 (27)	0.055

*GAF, Global Assessment of Functioning, symptom (S) and functioning (F) subscales; GSI/BSI, Global Severity Index from the Brief Symptom Inventory; DES, Dissociation Experience Scale*.

**Figure 6:**
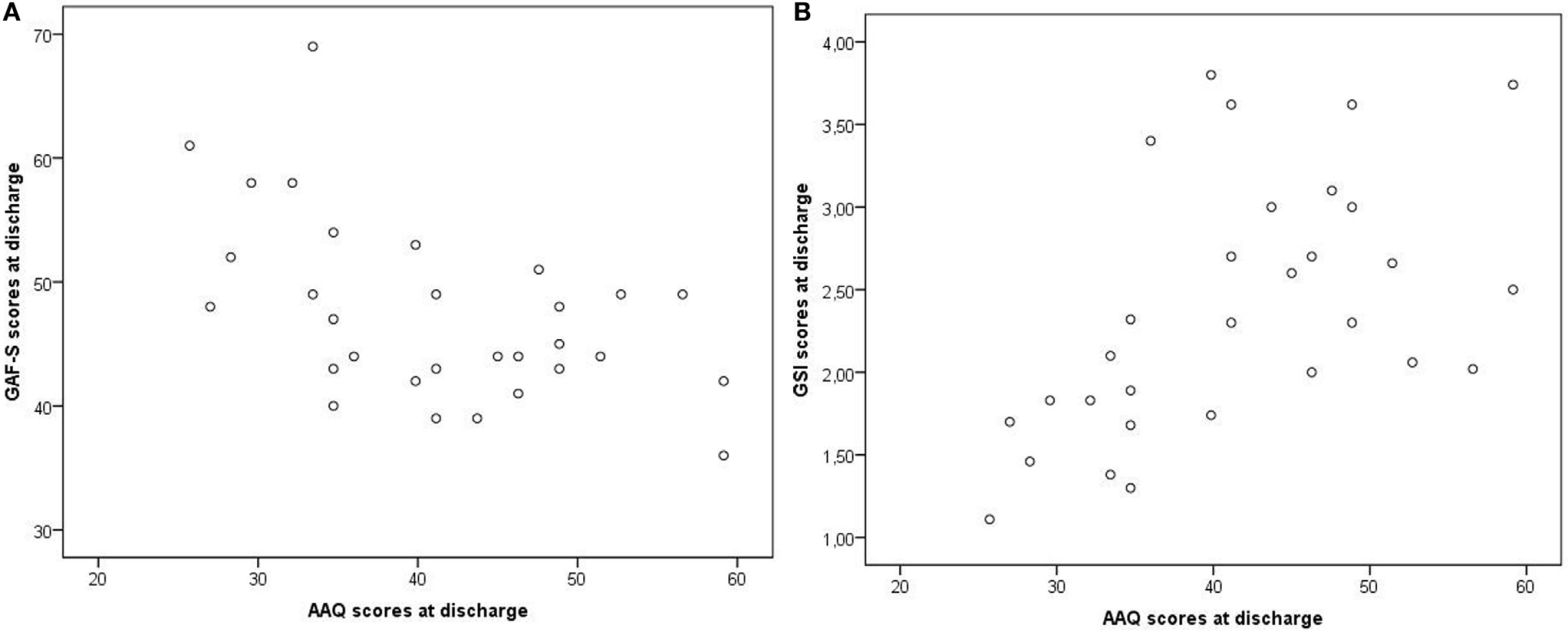
**Examples of correlations at discharge: AAQ vs. GAF-S and GSI**. AAQ, Acceptance and Action Questionnaire; GSI, Global Severity Index from the Brief Symptom Inventory; GAF-S, Global Assessment of Functioning, symptom subscale. Correlations: **(A)** AAQ vs. GAF-S, *r* = −0.52. **(B)** AAQ vs. GSI, *r* = 0.54.

At discharge, low dissociation (measured with DES) and low EA (measured with AAQ) were moderately correlated with high scores on GAF-F and GAF-S (less symptoms and better functioning) and with low scores on GSI (less symptoms), *p* values ≤ 0.031 (Table 5). The correlations were not due to outliers, as illustrated in Figures 6A,B for AAQ vs. GAF-S and GSI.

**Table 5:** **Associations of dissociation (DES) and EA (AAQ) with GAF and GSI at discharge**.

	GAF-S	GAF-F	GSI
DES	*r* = −0.40; *p* = 0.031	*r* = −0.42; *p* = 0.025	*r* = 0.52; *p* = 0.004
AAQ	*r* = −0.52; *p* = 0.003	*r* = −0.49; *p* = 0.006	*r* = 0.54; *p* = 0002

*GAF, Global Assessment of Functioning, symptom (S) and functioning (F) subscales; GSI/BSI, Global Severity Index from the Brief Symptom Inventory; DES, Dissociation Experience Scale*.

Time in BET was associated with changes in GAF-S, *r* = 0.44, *p* = 0.006, and GAF-F, *r* = 0.47, *p* = 0.003, but not with changes in GSI, DES, or AAQ, *p* values >0.60. When including degree of exposure in addition to time in BET as predictor of changes in GAF scores, in linear regression analysis, effects were seen for both predictors; GAF-S—time in BET, *t* = 3.5, β = 0.47, *p* = 0.001, degree of exposure, *t* = 2.9, β = 0.40, *p* = 0.006; GAF-F—time in BET, *t* = 4.0, β = 0.51, *p* < 0.001, degree of exposure, *t* = 3.6, β = 0.46, *p* = 0.001. The Durbin–Watson coefficients were normal, *d* = 2.01 for GAF-S and *d* = 1.88 for GAF-F, indicating no autocorrelation between residuals.

### Patient Predictors of Improvement

In a final series of *t*-tests and correlation analysis, we tested whether patient characteristics at baseline were associated with improvement at discharge. *t*-tests revealed no associations between 2 broad diagnostic categories (15 patients with psychosis vs. 23 patients without psychosis) and changes in any of the outcome measures, *p* values > 0.20. In contrast, the patients with the lowest scores on the outcome measures at enrollment to BET tended to have the largest improvements on the same measure at discharge. This was evident for GAF-S, *r* = −0.78, *p* < 0.001, GAF-F, *r* = −0.72, *p* < 0.001, and DES, *r* = −0.53, *p* = 0.003, with similar trends for GSI, *r* = −0.35, *p* = 0.06, and AAQ, *r* = −0.30, *p* = 0.11.

## Discussion

Patients who previously had not responded to repetitive treatment attempts showed improved symptoms and functioning after inpatient treatment with BET. The following pattern of improvements was observed across diagnostic categories. At discharge compared to enrollment, reduced symptoms were evident as higher scores on GAF-S and lower scores on BSI and DES, whereas improved functioning was evident as increased scores on GAF-F. Lower scores on AAQ at discharge compared to enrollment indicated reduced EA. Effect sizes were large, ranging from 0.76 for DES to 0.93 for GSI, 1.42 for GAF-F, 1.47 for AAQ, and 1.56 for GAF-S. In addition, the patients used less regular medications, i.e., antiepileptic, antipsychotic, anxiolytic, and antidepressant, at discharge than at treatment enrollment, with no concomitant increases in the use of alcohol or drugs. Patient improvement across treatment was associated with successful completion of the exposure component of BET, reduction in EA, high symptom levels and a low level of functioning at treatment start, and the duration of time in BET. At discharge, patients with the lowest levels of dissociation and EA tended to have the highest level of functioning (GAF-F) and the lowest level of symptoms (GAF-S and GSI).

One possible explanation to the improvements we observed is that patients were protected against the hassles and stresses of daily life while staying at the inpatient ward. In line with this possibility, longer time in BET was associated with stronger improvements in GAF scores. While protection against life stress may have played a role, regression analyses indicated that this was not the entire answer. Even if time in BET was the best predictor, independent effects on changes in GAF scores were seen for the degree to which the patients completed the exposure component of BET. Three other observations were consistent with the notion that BET had contributed to the patients’ improvements. First, the degree of completion of the exposure component, but not time in BET, also was associated with improvements on BSI and DES. Second, patients who underwent highest reduction in EA also had more improvements on GAF-S and GAF-F. Third, at discharge, patients with lowest EA and the lowest degree of dissociation tended to have the best scores on GAF and GSI.

### Effects of Second-Order Change Interventions

Significant reductions of dissociation (DES) and EA (AAQ), and these measures’ relationship to the other outcome parameters in the study, indicate that habitual avoidance to a large extent had been replaced with self-exposure and acceptance. The combination of findings may support the notion that second-order change interventions and improvement of psychological flexibility may be a feasible alternative to treatments that directly focus on symptom reduction in this patient population (26, 27). While we are aware of no comparative evidence for this conclusion regarding our patient group, changes in EA have been associated with symptom improvement in less ill patients. Hayes et al. (27) found that higher levels of EA were associated with a lower quality of life and higher levels of general psychopathology. In US samples, upper quartile scores on the 9-item version of the AAQ that we used have been reported at 42 in clinical samples drawn from people in outpatient psychotherapy with mild-to-moderate problems, and at 38 for non-clinical populations (undergraduate students) (27). Average AAQ scores in our patient group decreased significantly from 52.5 at BET enrollment to 41.4 at discharge. With the reservation that normative Norwegian data are lacking on the AAQ, our AAQ data indicate that while the BET patients at enrollment exhibited EA at levels associated with extremely high symptom loads and severe psychosocial problems, they presented with only mild-to-moderate psychological difficulties at discharge.

Since the goal of BET is to enable patients to cope with aversive and stressful inner experiences, treatment success would mean a reduced need for medication. The results show significant reductions in four of five medication categories, with a similar trend for the fifth category. At discharge, the patients had not compensated for reduced medications with increased use of alcohol or illegal drugs. This underscores the interpretation that BET facilitated second-order change processes and that there was no contribution from medications to the observed improvements.

The reduced EA and increased ability to self-regulate may indicate that BET has enhanced empowerment and autonomy. If this is correct, the patients through BET have attained ownership to tools that may lead to further improvement after discharge. Increased emotional and cognitive self-regulation may have reduced the patients’ emotional reactivity and increased their ability to handle negative emotions, relate to stress, choose from alternatives, make plans, and control their own behavior in a flexible manner (54). The underlying neuropsychological changes may include an increased capability to utilize frontal cortical systems to maintain cognitive control in situations with activation of subcortical emotion structures such as the amygdala (55, 56).

Exposure therapy is widely accepted as the treatment of choice for phobic conditions (57), and exposure is assumed to be the core psychotherapeutic change mechanism in BET. The results reveal that the patients who involved themselves in systematic graded exposure and flooding (high degree of exposure) benefited significantly more from treatment than those who did not (low degree of exposure). This may indicate that the specific component of basal exposure significantly contributed to the observed positive treatment responses. As a psychotherapeutic intervention, basal exposure is administrated as an inhibitory learning procedure (58). In contrast to habituation models of exposure (41), the ambition is not fear reduction but violation of expectations. By varying contextual cues both within and across the trials of exposure, BET seeks to enhance fear tolerance and generalize and consolidate the effects of exposure (58). However, it cannot be ruled out that the mechanism of habituation may have contributed to the indicated results.

Exposure depends on another treatment component, the establishment of a sufficiently strong and stable working alliance (59–61). A recent study by Hammer et al. (32) indicated that implementation of CER was followed by a significant reduction in the use of force as measured by number of resolutions. Being a comprehensive contextual intervention founded in a distinct therapeutic stance, the CER strategy may facilitate both extinction of behavioral disturbances and promote motivation for and willingness to engage in exposure therapy.

### Reconsidering “Treatment Resistance” in the Patient Group

The common treatment response across diagnostic categories, and the relatively better and diagnostically independent response among patients who presented with the lowest GAF scores at treatment start, may seem counterintuitive. The results may reflect and underscore the transdiagnostic qualities of BET and the model’s capacity to reach low-functioning patients. Such findings raise the question of whether the term “treatment resistant” is adequate to describe the patient group in this study. Instead, it is possible that a mismatch exists between the patients’ needs and the way they generally are met in specialized mental health care. One obstacle may be found in the clinical complexity of the patient group, where DSM and ICD diagnoses seem to be of limited value as tools to guide planning and implementation of individual treatments (26, 62, 63). The result may be what some BET patients have noted; throughout their long and turbulent histories in mental health care, they either have been given responsibility they were unable to handle or they have been subjected to a guardianship characterized by over-protective, external regulation defined as “necessary psychiatric care” (64–66). Failed treatment may, independent of what the cause has been, make health professionals start blaming and punishing the patient for the incompetence of the treatment (67, 68). An unfortunate consequence could be the too often frequent use of coercive measures such as forced medication, seclusion, and physical and mechanical restraints. The challenge may be to prevent and counter the development of marginalizing interaction patterns between patients and health-care professionals. By defining the problem this way, one solution is to redirect the focus of interventions to *the context that instigates* persevering dysfunctional behaviors on the part of the patient. By attributing causes of treatment resistance to the dynamics of the situations in which behaviors evolve, health professionals may be provided with tools to counteract and reverse marginalizing processes (32, 66).

### Strengths and Limitations

Strengths of the study include the use of both third person and first person measures of patient changes, showing comparable improvements in symptoms across treatment. Another strength is the within-group associations of therapeutic components and processes with the outcome measures that were consistent with basic assumptions in BET. The major limitation of this first evaluation study of BET is the naturalistic study design, with its lack of control group. While this limits the possibility to conclude about the effects of BET, the findings are still encouraging for launching a subsequent controlled prospective study, which we are currently planning. Another problem is that the apparent lack of prior studies related to this patient group precludes the comparison of our results with prior evidence. Moreover, the study included predominantly female patients. It is not clear whether the findings can be generalized to treatment-resistant, low-functioning male patients.

## Conclusion

Time-series data indicate that patients with severe and composite mental disorders who previously have appeared treatment resistant may benefit from BET in a hospital setting. Most importantly, the apparent success of BET indicates the possibility of recovery for these patients by ward-integrated psychotherapeutic means, providing an alternative to conventional treatments. While the evaluation data are promising, subsequent controlled studies are needed to establish whether BET is an effective treatment approach for the target patient group.

## Author Contributions

All authors contributed to study design, interpreting the data, and revising the manuscript. DH had the lead in writing the introduction and discussion parts of the manuscript. RF performed the statistical analysis and had the lead in writing up the methods and results sections. JH supervised data acquisition. All authors approved the final version of the manuscript.

## Conflict of Interest Statement

DH and JH have received payment for presentations of Basal Exposure Therapy. RF reports no conflict of interest.

## Footnotes

^1^Hayes SC, Bissett RT, Strosahl KD, Wilson KD, Pistorello J, Dykstra TA. *Psychometric Properties of the Acceptance and Action Questionnaire (AAQ)* (2000). Unpublished manuscript.
